# Anomalie d’émergence radiculaire par racine conjointe S1: à propos d'un cas

**DOI:** 10.11604/pamj.2015.20.166.6086

**Published:** 2015-02-23

**Authors:** Yannick Canton Kessely, Maguette Gaye Sakho, Alidji Fondo, Akerey Diop Daisy, Aley Thiam, Youssoupha Sakho

**Affiliations:** 1 Service de Neurochirurgie de l'Hôpital Général de Grand Yoff, Dakar, Sénégal

**Keywords:** Hernie discale, chirurgie, anomalie radiculaire, herniated Disc, surgery, root anomaly

## Abstract

De découverte per-opératoire pour hernie discale, les anomalies d’émergence radiculaires constituent une entité rare. La présentation clinique est généralement identique à celle d'une radiculopathie. Nous rapportons le cas d'un patient âgé de 35 ans présentant une sciatique droite S1 hyperalgique rebelle aux multiples médications. L'IRM du rachis lombo-sacré avait mis en évidence une discopathie protrusive de petit volume en L5 S1 droite et un aspect de grosse racine ou de kyste de Tarlov au niveau de l’émergence droite de S1. Une fenestration inter lamaire L5-S1 droite avec une ablation du ligament jaune a montré une émergence radiculaire double au niveau de l'espace retro-discal. Un geste de foraminostomie a été réalisé sans discectomie. L’évolution a très favorable avec une rétrocession dès le lendemain. L'analyse pré-opératoire fine et rigoureuse de l'imagerie est indispensable. Une bonne libération améliore l’état clinique du patient. Y penser en cas de sciatique hyperalgique sans Lasègue.

## Introduction

De découverte le plus souvent per-opératoire, les anomalies d’émergence radiculaire par racines conjointes peuvent constituer une cause d’échec de la chirurgie vertèbro-discale. Elles sont définies comme deux racines nerveuses adjacentes qui partagent une enveloppe durale commune à un moment donné au cours de leur trajet [1]. Fréquemment unilatérales, des cas bilatéraux ont été notés. Connue depuis l’ère de la saccoradiculographie, elle a bénéficié actuellement d'une meilleure analyse avec l'apport de l'imagerie par résonance magnétique (IRM). De récents progrès de l'IRM semblent permettre d'identifier avec plus de précisions cette anomalie avant chirurgie [2]. Nous rapportons un cas de racines conjointes de découverte per-opératoire lors d'une chirurgie pratiquée pour hernie discale.

## Patient et observation

**Figure 1:**
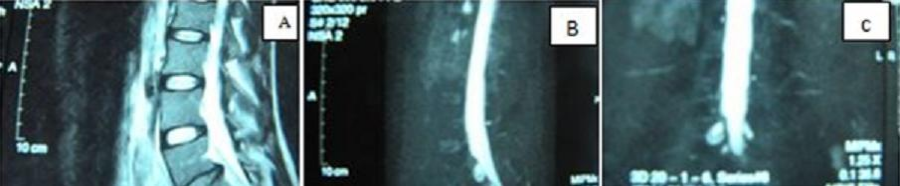
(A) IRM en coupe sagittale séquence T2 montrant une protrusion discale en L5S1; (B) IRM en T2 de profil; (C) TDM reconstruction coronale montrant une grosse racine à droite

**Figure 2:**
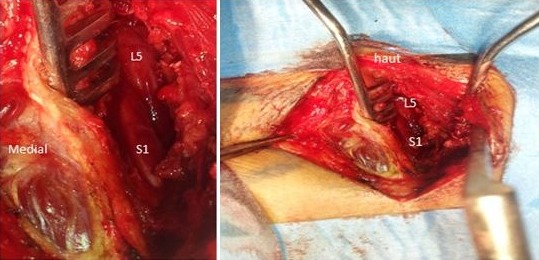
Vue per-opératoire. L5: racines L5; S1: racine S1

Il s'agit d'un patient âgé de 35 ans présentant depuis 3 mois une sciatique droite S1 hyperalgique rebelle aux multiples médications (paracétamol; tramadol; AINS; anti dépresseurs tricyclique; clobazepam; prégabaline). A l'interrogatoire, aucune notion d'effort ou de traumatisme n'a été notée et le patient n'avait pas de passé de lombalgie. L'examen clinique a noté une perte pondérale de moyenne abondance. L'examen était quasi impossible du fait d'une hyperpathie plantaire confinant le patient au lit, une sciatique droite de topographie S1 sans lombalgie, avec des phénomènes neuropathiques de type brulure; décharges électriques; allodynie; sensation de froid douloureux, le patient ne pouvant poser le pied droit au sol. Une douleur aux points de Valleix a été mise en évidence. Il n'y avait ni raideur rachidienne ni un déficit moteur, mais une hypoesthésie plantaire droite. Les reflexes ostéo-tendineux n'avaient pu être recherchés à cause de la douleur, cependant un signe de Lasègue avait été noté en fin de course. L'IRM du rachis lombo-sacré avait mis en évidence une discopathie protrusive de petit volume en L5 S1 droite et un aspect de grosse racine ou de kyste de Tarlov au niveau de l’émergence droite de S1 (Figure 1). Une fenestration inter lamaire L5-S1 droite avec une ablation du ligament jaune a permis de découvrir la présence d'une émergence radiculaire double au niveau de l'espace retro-discal, les 2 racines cheminant côte à côte vers le récessus latéral (Figure 2). La racine médiale avait un aspect congestif et était difficile à mobiliser. Le récessus latéral était sténosé. Ces racines conjointes occasionnaient un conflit d'espace. Un geste de foraminostomie a été réalisé sans discectomie. Les suites opératoires furent simples avec rétrocession rapide des phénomènes algiques dès le lendemain ainsi qu'une amélioration notable, une déambulation et une diminution nette de l'allodynie plantaire. Le patient a été exéaté le 4ème jour avec une disparition de la sciatalgie.

## Discussion

**Figure 3:**
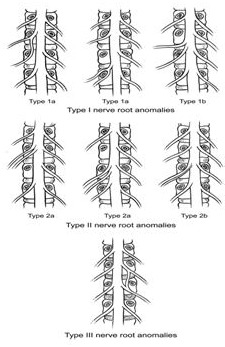
Classification de Neidre et MacNab

Les anomalies des racines nerveuses sont décrites dans 8,5% à 30% sur les études cadavériques, et dans 2 à 17,3% sur des études radiographiques [3]. Les racines L5-S1 et S1-S2 sont les plus concernées. Plusieurs classifications ont été proposées pour cette pathologie depuis la première description anatomique faite en 1949 par Zagnoni. Il y a la classification de Cannon et al, de Neidre ac Nab, de Postacchini de Kadish et Simmons et celle de Kikuchi [4]. Le type I de la classification de Neidre et MacNab est le plus fréquent, et le type III le plus rare. Notre patient est du type IIb de la classification de Neidre et MacNab et l'anomalie porte sur les racines L5 et S1 (Figure 3). Le type 1 de la classification concerne des racines partageant le même fourreau dural et ayant une émergence différente au niveau des trous de conjugaison. Le type II concerne les racines partageant la même émergence; et le type III des racines ayant des émergences différentes mais reliées par une anomalie radiculaire.

Souvent asymptomatiques, l'adjonction des phénomènes dégénératifs responsables d'une compression tels que la hernie discale, une sténose latérale du récessus, ou un spondylolisthesis [3] peuvent rendre symptomatiques les anomalies des racines nerveuses. Comme ce fut le cas de notre patient, il y avait une sténose du récessus latéral pouvant expliquer la symptomatologie bruyante. Cependant, pour d'autres auteurs [5], le caractère symptomatique peut exister en dehors de tout mécanisme compressif et se traduire par une lombosciatalgie mal systématisée, intermittente et parfois biradiculaire. Le symptôme radiculaire avec douleur au repos est plus fréquent chez les patients ayant une hernie discale [3]. Ceci est confirmé par l’état de notre patient qui avait une hernie discale en L5S1. Selon Decq et al [5], la symptomatologie de ces anomalies serait liée juste à la mobilité limitée et non nécessairement à tout élément compressif. Le caractère intermittent de la sciatique et l'absence de signe de Lasègue pourraient orienter le diagnostic; or la présence du signe de Lasègue ne fait pas l'unanimité. Ainsi, James White et al [6] ont rapporté au cours de leur étude 40% de leur patients n'avaient pas un signe de Lasègue, de même que Taghipour [7] chez 36% de ses patients. Un Lasègue en fin de course a été objectivé chez notre patient. Actuellement, l'IRM représente l'examen de choix permettant d'objectiver les différents types d'anomalie même ceux dont le diagnostic est difficile à faire au myeloscanner, notamment en coupes coronales, sequences T1 et T2. Selon Lotan et al [3] le diagnostic imagérique à la myélographie, au scanner ou à l'IRM en préopératoire reste un problème malgré l'amélioration des moyens d'investigation. Pour Younes [8], le scanner est moins contributif au diagnostic; mais il peut parfois montrer l'anomalie sous forme d'une grosse racine, d'une racine bilobée ou dédoublée au niveau de récessus latéral. Pour notre patient, l'IRM a plutôt montré un aspect de grosse racine pris pour un kyste de Tarlov. Des confusions sont possibles entre la racine et un fragment discal au myeloscanner [5]. Kang et al [9] ont décrit le sagittal shoulder sign: « Le signe de l’épaule » représentant une structure verticale reliant deux racines consécutives et recouvrant une hernie discale. Malgré la description de certains signes à l'IRM en coupe axiale tels que l'asymétrie du coin antérolatéral: « The corner sign »; le croissant graisseux extradural: le « fat crescent signe », les erreurs diagnostics sont encore possibles car il n'y a pas de signe pathognomonique de racines conjointes [10].

Le traitement de la sciatique avec anomalies des racines nerveuses est le même que celui d'une sciatique commune [8]. Ceci a été le cas du patient. Différentes approches de prise en charge ont été proposées. Ainsi pour les cas asymptomatiques ou de découverte fortuite Artico et al, ne préconisent aucun traitement [10]. La chirurgie de cette lésion est controversée. Le traitement chirurgical consiste à pratiquer une libération large des racines (foraminectomie, arthrectomie, voire une pédiculectomie) et une résection d'une éventuelle hernie associée [8]. Une foraminectomie seule a été faite dans notre cas sans discetomie car la mobilisation de la racine médiale était difficile. Pour Taghipour [7], la discectomie seule est insatisfaisante. Pour les cas symptomatiques sans hernie discale, une infiltration de corticoïdes peut être faite. Les résultats sont meilleurs en cas d'un élément compressif associé et lorsque le diagnostic est réalisé en préopératoire.

## Conclusion

Dans le cadre de la chirurgie endoscopique, la plupart des accidents sont liés à la méconnaissance des racines conjointes. L'analyse fine de l'imagerie avant chirurgie est indispensable pour minimiser les découvertes peropératoires et éviter ainsi un traumatisme ou un échec chirurgical. Une bonne libération améliore l’état clinique du patient. Y penser en cas de sciatique hyperalgique sans Lasègue.
